# New drug-delivery balloon catheter for easy and fast injection of triamcinolone after esophageal endoscopic submucosal dissection

**DOI:** 10.1055/a-2353-5973

**Published:** 2024-07-26

**Authors:** Takashi Hirose, Hiroyuki Shibata, Kazuhiro Furukawa, Takeshi Yamamura, Takuya Ishikawa, Masanao Nakamura, Hiroki Kawashima

**Affiliations:** 1 Department of Endoscopy, Nagoya University Hospital, Nagoya, Japan; 2 Department of Gastroenterology and Hepatology, Nagoya University Graduate School of Medicine, Nagoya, Japan

Esophageal endoscopic submucosal dissection (ESD) is a very common and useful endoscopic treatment for superficial esophageal cancer 1 . The most common delayed adverse event of esophageal ESD for extensive lesions is stenosis 2 . Local injection of triamcinolone is the most common form of stenosis prevention 3 , and the method using a needle is a rather complicated and time-consuming procedure. A new drug-delivery balloon catheter is a device with multiple micro holes on its surface that eject fluid from the balloon when the balloon pressure rises.

A man in his 60s underwent ESD for semiperipheral superficial esophageal cancer in the upper thoracic esophagus ( Fig. 1 ). The resected pathology was squamous cell carcinoma, and curative resection was obtained. After resection, the mucosal defect extended to 7/8 of the circumference ( Fig. 2 ), and triamcinolone 40 mg was injected into the post-treatment ulcer using the new drug-delivery balloon catheter ( Video 1 ).

**Fig. 1:**
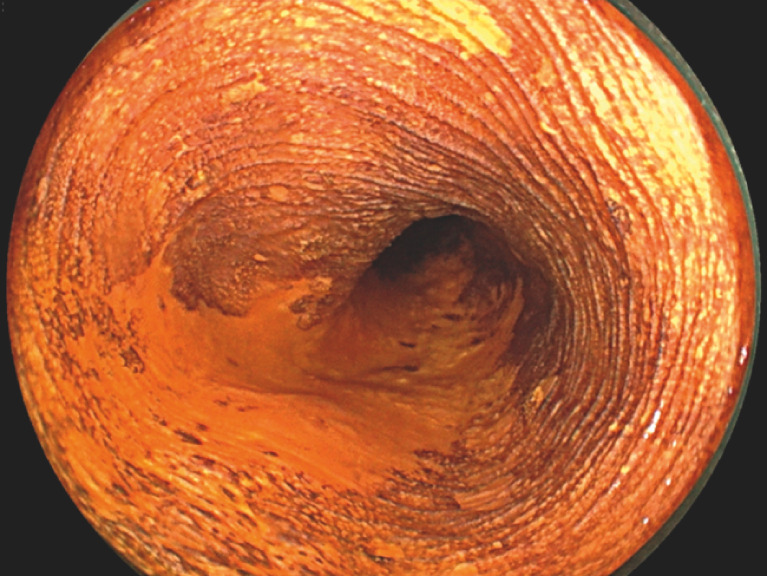
Semiperipheral superficial esophageal cancer in the upper thoracic esophagus.

**Fig. 2:**
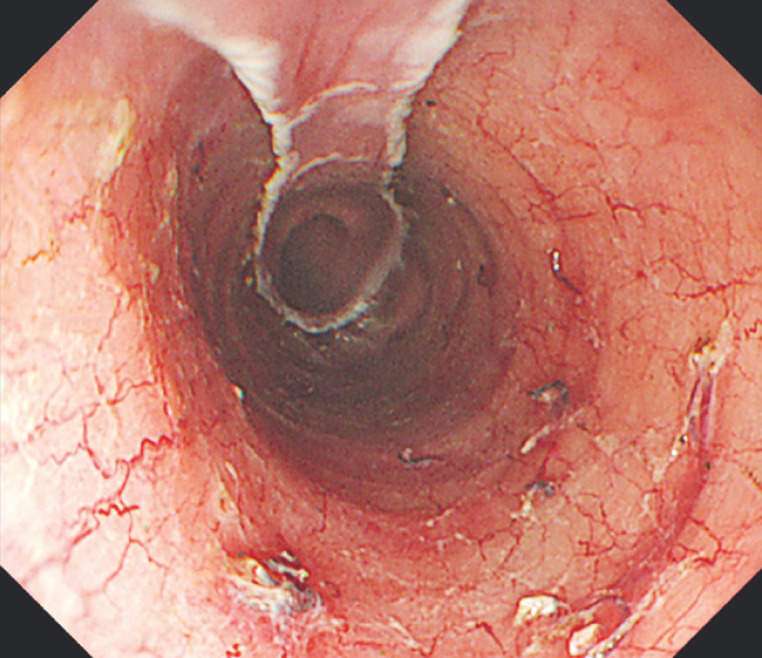
After endoscopic resection, the mucosal defect extended to 7/8 of the circumference.

After resection, the mucosal defect extended to 7/8 of the circumference, and triamcinolone 40 mg was injected into the post-treatment ulcer using the new drug-delivery balloon catheter.Video 1

The procedure was completed in only a few minutes, and was very safe and simple. Post-procedure observation showed that triamcinolone was injected evenly and firmly into the ulcer ( Fig. 3 ).

**Fig. 3:**
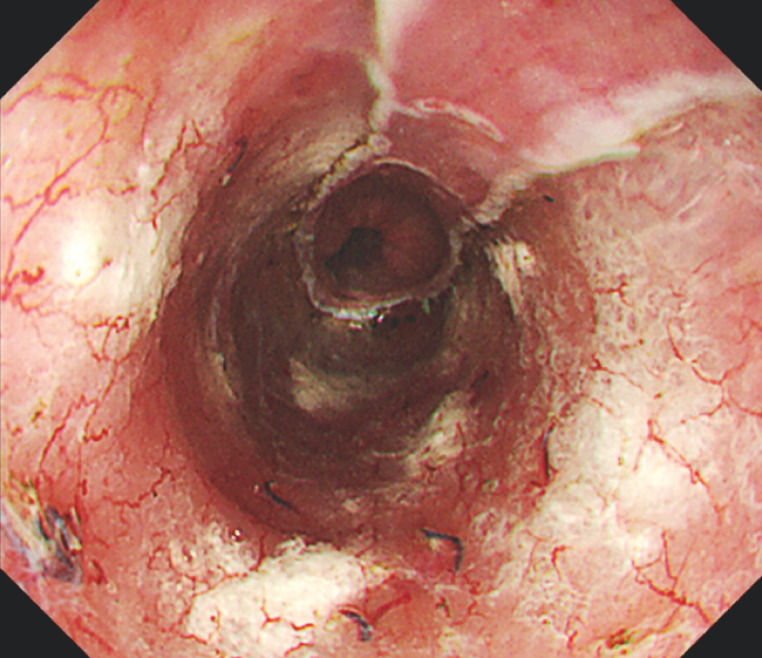
Post-procedure observation showed that triamcinolone was injected evenly and firmly into the ulcer.

A new drug-delivery balloon catheter allows for safe, simple, and quick triamcinolone injection after esophageal ESD.

Endoscopy_UCTN_Code_TTT_1AO_2AG_3AD

## Notes

Endoscopy E-Videos https://eref.thieme.de/e-videos
*E-Videos* is an open access online section of the journal *Endoscopy* , reporting on interesting cases and new techniques in gastroenterological endoscopy. All papers include a high-quality video and are published with a Creative Commons CC-BY license. Endoscopy E-Videos qualify for HINARI discounts and waivers and eligibility is automatically checked during the submission process. We grant 100% waivers to articles whose corresponding authors are based in Group A countries and 50% waivers to those who are based in Group B countries as classified by Research4Life (see: https://www.research4life.org/access/eligibility/ ). This section has its own submission website at https://mc.manuscriptcentral.com/e-videos .
